# Aspects of Solvent Chemistry for Calcium Hydroxide Medicaments

**DOI:** 10.3390/ma10101219

**Published:** 2017-10-23

**Authors:** Basil Athanassiadis, Laurence J. Walsh

**Affiliations:** 1 Private dental practice, Annerley, QLD 4013, Australia; basildent@bigpond.com; 2 School of Dentistry, University of Queensland, Herston, Brisbane, QLD 4004, Australia

**Keywords:** endodontics, medicaments, calcium hydroxide, polyethylene glycol, alkalinity, disinfection

## Abstract

Calcium hydroxide pastes have been used in endodontics since 1947. Most current calcium hydroxide endodontic pastes use water as the vehicle, which limits the dissolution of calcium hydroxide that can be achieved and, thereby, the maximum pH that can be achieved within the root canal system. Using polyethylene glycol as a solvent, rather than water, can achieve an increase in hydroxyl ions release compared to water or saline. By adopting non-aqueous solvents such as the polyethylene glycols (PEG), greater dissolution and faster hydroxyl ion release can be achieved, leading to enhanced antimicrobial actions, and other improvements in performance and biocompatibility.

## 1. Introduction

The pulpo-dentine complex of a tooth is covered by enamel or cementum, which protects it from the oral microflora. The integrity of this protection can be breached by many processes, including dental caries, trauma to the tooth, and cracks, which then allows infection of the pulpo-dentine complex to occur, resulting in most cases in pulp and periapical inflammatory diseases [1,2]. Bacteria and fungi from the human oral microbiome can invade into the root canal system of teeth. Penetration of bacteria into dentinal tubules can range up to 300 microns [3,4,5]. Microorganisms organized into biofilms can also be found around the apex on the external root surface [6].

Clinical protocols for multiple visit endodontics include the routine use of medicament pastes placed into the canal to achieve disinfection. This clinical approach recognizes that there are many areas of the root canal system that are not instrumented by hand or powered instruments, and which may not be reached by conventional irrigation methods. This is particularly the case in the apical third of the canal, where canal walls after debridement are often found to be contaminated by debris, and where there are many regions that have not been instrumented [7,8,9].

Placing an antimicrobial agent into the canal allows a time period of one week or more for the active ingredients to diffuse from the paste through the root canal system and into the radicular dentine, reaching microorganisms that may be residing in biofilms in protected areas or that are sequestered deep within dentine tubules [10,11]. The need for an intracanal antimicrobial medication is greater in clinical cases where the bacteria and/or fungi present are resistant to routine treatment. Such microorganisms, when situated within dentinal tubules, constitute a reservoir from which re-infection of the root canal may occur. Thus, the active ingredients released from endodontic medicaments must be able to penetrate through biofilms, into the root and particular into dentinal tubules to kill bacteria and fungi located within the tubules [12,13,14].

The most commonly used agent for inter-visit dressing is calcium hydroxide. In addition to its use for inter-visit dressing, it is also used for apexification and direct pulp capping [15,16,17,18]. These clinical applications exploit the ability of calcium hydroxide to stimulate hard tissue formation, as well as to exert antimicrobial actions. Released calcium ions play important roles in cell stimulation, migration and proliferation, as well as in mineralization and hard tissue repair [19,20,21].

Hydroxyl ion release from calcium hydroxide is responsible for the key attributes of broad spectrum antimicrobial activity, penetration into biofilms, inhibition of endotoxins, and dissolution of organic tissues [21]. The lethal effects of hydroxyl ions are mediated by several mechanisms, including damage to the microbial cytoplasmic membranes, damage to DNA, inhibition of DNA replication, and suppression of enzyme activity, and disruption of cellular metabolism [22]. Calcium hydroxide materials also kill remaining micro-organisms in the root canal system by withholding substrates for growth and by limiting space for multiplication [22]. Calcium hydroxide medicaments also act as a physical barrier to prevents the ingress of bacteria into the root canal system [22]. While most microorganisms found in the root canal can be inactivated by calcium hydroxide, *Enterococcus faecalis* and *Candida albicans* are well known to be resistant to actions of saturated aqueous solutions of calcium hydroxide [3,23,24].

## 2. Solvents and Their Effects on Solubility

Since calcium hydroxide kills bacteria through the effects of hydroxyl ions, the efficacy of any given calcium hydroxide product varies according to on the availability of these ions in solution, which in turn reflects the nature of the solvent used [25]. Most existing commercial calcium hydroxide pastes use vehicles of water, since this was the original formulation dating back to the 1920s. Other agents have been mixed into the water, including glycerin or polyethylene glycol (PEG), both of which serve as thickeners and increase the viscosity [26].

More recently, non-aqueous preparations based on PEG 400 have also been developed (Table 1). PEG 400 is a colourless, water-soluble and hygroscopic polymer that is miscible with water in all proportions, so it can be used as a solvent in its own right or can be mixed with water. PEG is classified as “Generally Recognised as Safe” and has high biocompatibility. As seen in Table 1, materials based on PEG solvents can achieve a pH which is above 12.4–12.6, the nominal limit for aqueous compositions. The combination of PEG 400 with PEG 3350 has been found to inhibit the growth of *Prevotella intermedia*, an endodontic and periodontal pathogen [27], as well as other Gram-negative bacteria [28].

The solubility of calcium hydroxide in water is low, being only 0.159 g/100 mL (0.16% by weight) at 25 °C. This reduces to 0.140 g/100 mL (0.14% by weight) at 40 °C, with an accompanying decrease of 0.033 pH unit/°C with increasing temperature [31]. When distilled water or saline is used as a solvent, the common ion effect operates, since water already contains some free hydroxyl ions. Considering the low solubility in water, most water-based calcium hydroxide endodontic pastes contain a large excess of calcium hydroxide, well beyond that which can be dissolved. This excess above the solubility limit will not elevate the hydroxyl ion release or pH which can be achieved, since no further material can dissolve. The undissolved material will, however, act as a thickener in the paste, and as hydroxyl ions leave the paste to diffuse into the surrounding environment of the tooth, the undissolved material can also act as a reservoir [31].

Compared to water, the solubility of calcium hydroxide is much higher in glycerin and in PEG [31]. These more viscous vehicles can be loaded with calcium hydroxide when then is a reservoir for prolonged release of hydroxyl ions, creating a highly alkaline environment which is not conducive to the survival of bacteria or fungi. It is thought that using a more viscous solvent could improve the clinical handling and slow the rate of hydroxyl ion release, allowing for less frequent re-dressing of the root canal system [32].

PEG 400 has been used as a thickening agent to improve the clinical handling of calcium hydroxide pastes [33,34,35]. Both glycerin and PEG allow greater dissolution of calcium hydroxide than water, giving enhanced release of hydroxyl ions [36,37,38]. Because of these actions, there is not a clear correlation between the weight loading of calcium hydroxide and the pH measured using a traditional electrode. As seen in Table 1, the product with the highest weight loading (DT Temp with 50% in water) is not the most alkaline; rather, the products with PEG 400 show greater measured alkalinity despite having a much lower total calcium hydroxide content.

PEG 400 is a suitable solvent for calcium hydroxide, and gives superior ion release into roots than a water solvent [31,39,40]. This can be attributed to the large number of ethylene oxide groups along its backbone, which allows PEG to form complexes with calcium ions, driving the dissociation of calcium hydroxide, and thus releasing more free hydroxyl ions [31]. From a clinical perspective, enhanced calcium hydroxide dissolution should allow the medicament to act over a longer period of time and reduce the frequency of re-dressing canals, particularly in cases where there has been long-standing periapical infection [41].

The alkaline effects achieved by calcium hydroxide are especially important when considering the effects on highly resistant bacteria and fungi that are likely to be present in persistent and refractory infections. *Enterococcus faecalis* is able to survive at pH 10, but not at pH 11.5–11.9 and above [13,42,43] These levels are difficult to achieve and to maintain in the root canal environment because of the buffering effects of dentine proteins [30,41]. In order to achieve disinfection of dentine, calcium hydroxide medicaments must resist inactivation by a number of organic components (including dentine collagens, dentine phosphoproteins, necrotic pulp tissue remnants and inflammatory exudate), inorganic components (apatites) and bacterial biofilms [30]. Saturated aqueous solutions of calcium hydroxide are much more prone to rapid inactivation by dentine buffering than medicament pastes which use viscous vehicles and have higher loadings of calcium hydroxide. A saturated aqueous solution lacks any reservoir capacity, unlike a medicament paste.

Achieving greater dissolution of calcium hydroxide in the solvent is essential for overcoming this buffering action and ensuring that high pH levels are sustained in the radicular dentine. After placement of a calcium hydroxide medicament paste into the root canal, the process of hydroxyl ion diffusion will begin, being more rapid in the cervical third of the root than in the apical third where the tubules are smaller and less numerous. The effects of the released hydroxyl ions can be seen on the inner root dentine (i.e., adjacent to the root canal) within a matter of hours, and the middle regions of the dentine over the following week, taking 2–3 weeks to reach peak pH levels [7,31,39,40,41].

In addition to dentine buffering of hydroxyl ions, another limiting action for calcium hydroxide is partial conversion to calcium carbonate, from contact with carbon dioxide or with carbonate ions. The formation of calcium carbonate attenuates the antimicrobial actions, since calcium carbonate has very low solubility, and it creates only a mildly alkaline pH of 8.0, which is too low to inactivate most bacteria. This conversion reaction happens mostly in the apical third of the canal [44].

## 3. Measurements of pH of Calcium Hydroxide Preparations and Their Interpretation

At 25 °C, the pH range in water is from 0 to 14. Each pH unit corresponds to a 59.16 mV change in electrode potential. When the temperature is higher than 25 °C, the sum of pH + pOH is slightly less than 14 due to a higher degree of ionization of water. Conversely, at low temperatures, pH + pOH is larger than 14 due to a lesser degree of ionization.

Freshly prepared solutions of calcium hydroxide in water show a typical pH of 12.4–12.7 [21,41]. If left to stand over time, the pH falls, as conversion to calcium carbonate occurs due to the presence of dissolved carbon dioxide gas and, thus, carbonic acid.

**Figure 1:**
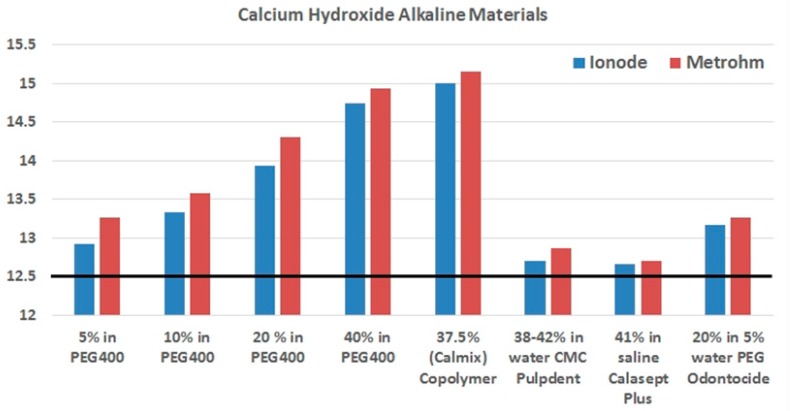
pH measurements using a KCl electrode (Ionode) (blue bars) and a tetraethylammonium bromide (TEABr) electrode (Metrohm Solvotrode)(model, company, city, country) (red bars) according to the percentage weight loading of calcium hydroxide in different solvents, comprising (from the left to the right) PEG 400, PEG 400 + PEG 3350 copolymer, water with carboxymethylcellulose (CMC), saline, and a mix of 5% water and PEG 400. Two commercial pastes are water based (Pulpdent, Calasept Plus), and two use PEG 400 (Odontocide, Calmix). The typical pH achieved for calcium hydroxide in water is shown by the horizontal black line. Data from Reference [31].

Just as with water, pH scales may also be defined in non-aqueous liquids at a particular temperature [45,46,47] (Table 2). It is possible to consider pH in non-aqueous fluids because the proton activity term is applicable to organic solvents as well as to water. While pH scales in non-aqueous fluids are equally valid as a measure of acidity and dissociation constants, a separate pH scale is required for each solvent, as the scale varies according to the solvent. This is particularly important for alkaline preparations. When strong bases are added, at room temperature, the upper pH limit of water is pH 14, while the upper pH limit in PEG 400 is 15.8, which is almost 2 pH units greater than water. This explains why a pH value of over 14 can be recorded for a PEG-based calcium hydroxide product, and how pH rises as more calcium hydroxide is added into PEG 400 (Figure 1). When measured with a KCl electrode (model, company, city, country), the pH of a 10% solution of calcium hydroxide in PEG 400 is 14.75 [31], which is two pH units above the maximum pH of 12.7 for freshly prepared solutions of calcium hydroxide in water [21,41].

Other upper pH limits that can be reached when strong bases are added include ethylene glycol pH 15.8, ammonia pH 32.5, and dimethyl sulphoxide pH 35.0 [49,50,51]. Likewise, the pH measurement scales in non-aqueous solutions will also show changes in lower limits when compared to water, and this will also occur when water is mixed with organic solvents [52]. The pH scale for an ethanol solvent is −4 to +16 pH, while for an acetone solvent it is −5 to +20 pH [52]. The reason for this change in the scale is that ethanol can act as both a proton donor and proton acceptor, which moderates changes in proton activity. In the case of acetone, it is neither a proton donor nor a proton acceptor, and so does not influence proton activity. This allows the upper and lower limits of the pH scale to be much further apart than is the case for water [53]. This concept, which is well known in the field of electrochemistry [49,50,51,52,53], has direct relevance to the discussion of pH measurements of calcium hydroxide in various solvents. Like acetone and many other hydrocarbons, the pH scale for a PEG solvent has an extended upper pH limit. This higher alkaline limit is one reason why PEG is a suitable material to use as solvent for calcium hydroxide products.

Finally, a range of technical issues arise when measuring the properties of highly alkaline materials with pH sensors. The most important of these is that traditional pH sensors are built with wetted materials designed for aqueous applications. Thus, when using non-aqueous solvents with water-free materials, a special electrode designed for non-aqueous pH measurement should be used. Electrodes designed specifically for non-aqueous solvents use 2% lithium chloride in ethanol as the reference electrolyte. When testing highly alkaline materials, the electrode solution used is tetraethylammonium bromide (TEABr) 0.4 mol/L in ethylene glycol. The same solutions are used to store the electrodes when not in use. Because of the slow speed of response of these electrodes, when PEG-based medicaments (such as Calmix or Odontocide) are assessed, up to 140 min is required before a stable recording is reached. Typical data from an evaluation of these products is shown in Table 3.

Data show the mean and standard deviation from five independent experiments. In this series, the ranking of medicaments from most alkaline to least alkaline was Calmix, Odontocide, Pulpdent and then Calasept Plus, regardless of which of the two electrodes was used. With the KCl electrode, all measured pH differences between materials were statistically significant (*p* < 0.001), except for Pulpdent versus Calasept Plus. In contrast, with the TEABr electrode, all differences between Calmix and other materials were statistically significant (*p* < 0.001), but there was no significant difference between the other three pastes. Differences in the readings between the two electrodes vary from 0.05 to 0.16 pH units. 

There is an extensive literature on the choice of electrodes for measuring non-aqueous fluids [54,55,56,57,58,59]. Methods have been developed to use semiconductor pH sensors for non-aqueous fluids. Ta_2_O_5_– and Si_3_N_4_–type pH–sensitive ion-sensitive field-effect transistors (ISFETs) have been used to measure pH in non-aqueous solutions, and were found to respond much faster than the conventional glass electrodes [54].

## 4. Conclusions

Calcium hydroxide is a widely used antimicrobial agent in endodontics. The solvent used for this agent influences both the physical and chemical properties of the material, including its viscosity and ion release pattern. A range of water-based materials have traditionally been used for calcium hydroxide medicament pastes, but there has also been interest in the use of alternative vehicles including glycerin and PEG, since these are better solvents and can enhance ion release. The concepts of ionic equilibria and pH in non-aqueous solvents that have been well documented in the electrochemistry literature can help explain how water-free solvents influence the properties of calcium hydroxide when dissolved into glycerin or PEG. Water-free medicaments may be used to achieve enhanced alkaline effects for inactivating microorganisms in the root canal.

## Figures and Tables

**Table 1:** Calcium hydroxide pastes used in endodontics.

Name	Ca(OH)_2_ Content and Vehicle	Measured pH
ApexCal^TM^	29% in water + PEG + glycerin	12.4
Calen^TM^	49.8% in PEG 400	N/A
Calasept Plus^TM^	41.1% in saline (water)	12.6
Calcipulpe^TM^	20% in water	11.8
Calmix^TM^	37.5% in PEG 400 + PEG 3350	15.0
DT Temp^TM^	50% in water	12.6
Odontocide^TM^	20% in PEG 400 + water	13.2
Pulpdent^TM^	42% in water	12.7
Ultracal XS^TM^	35% in water	12.5

N/A = not available. Data on pH taken from References [21,27,29,30,31].

**Table 2:** pH windows in various solvents.

Solvent	Lower Limit	Upper Limit
Water	0	14
Sulfolane	−10	31
Methanol	1.8	17.2
Ammonia	18	32.5
Ethanol	−4	16
Acetone	−5	20

Based on Reference [48].

**Table 3:** pH assessments using conventional and non-aqueous electrodes.

Product	KCl Electrode	TEABr Electrode
Pulpdent	12.706 (0.006)	12.862 (0.008)
Calasept Plus	12.662 (0.017)	12.710 (0.012)
Odontocide	13.170 (0.025)	13.062 (0.450)
Calmix	14.996 (0.010)	15.162 (0.108)
